# Supervised Machine Learning Models to Identify Early-Stage Symptoms of SARS-CoV-2

**DOI:** 10.3390/s23010040

**Published:** 2022-12-21

**Authors:** Elias Dritsas, Maria Trigka

**Affiliations:** Department of Computer Engineering and Informatics, University of Patras, 26504 Patras, Greece

**Keywords:** healthcare, SARS-CoV-2, machine learning, prediction, data analysis

## Abstract

The coronavirus disease (COVID-19) pandemic was caused by the SARS-CoV-2 virus and began in December 2019. The virus was first reported in the Wuhan region of China. It is a new strain of coronavirus that until then had not been isolated in humans. In severe cases, pneumonia, acute respiratory distress syndrome, multiple organ failure or even death may occur. Now, the existence of vaccines, antiviral drugs and the appropriate treatment are allies in the confrontation of the disease. In the present research work, we utilized supervised Machine Learning (ML) models to determine early-stage symptoms of SARS-CoV-2 occurrence. For this purpose, we experimented with several ML models, and the results showed that the ensemble model, namely Stacking, outperformed the others, achieving an Accuracy, Precision, Recall and F-Measure equal to 90.9% and an Area Under Curve (AUC) of 96.4%.

## 1. Introduction

Coronaviruses are a group of viruses that often cause generally mild respiratory infections in humans and animals. Most people are infected with coronaviruses at least once in their lives, having mild to moderate symptoms of the common cold. Rarely does a coronavirus mutate and spread from animals to humans, as has happened with SARS-CoV-2 today but also in the past with the SARS (2003) and MERS (2012) viruses. When a new virus infects humans, no one is immune, and everyone can become infected. This wide spread of the virus is also the reason why it has caused global concern, and the World Health Organization (WHO) declared the COVID-19 pandemic on March 2020 [1,2,3].

Respiratory droplets and aerosols produced by sneezing, coughing and direct or close contact with other people (usually less than two meters) are some common conditions under which the new strain of coronavirus can be spread from person to person causing them to become infected when they touch their nose, mouth or eyes. The virus’s survival is dependent on the material of the surface. In particular, it can survive for several hours on copper or cardboard and/or up to a few days on plastic or stainless steel. The average time between exposure to the virus and the onset of symptoms, known as the incubation period, for COVID-19, is currently estimated to be 5 to 6 days, or generally 1 to 14 days. According to an analysis made by the WHO, each patient may infect 1.4–2.5 other people (compared to the seasonal flu, where each patient infects an average of 1.3 other people) [4,5,6].

COVID-19 disease varies greatly in severity. There may be a complete absence of symptoms (asymptomatic patients), or symptoms such as fever, cough, sore throat, change or loss of taste and/or smell, general weakness, diarrhea, fatigue, and muscle pain may occur. In severe cases, symptoms may include severe lung infection, generalized infection and inflammatory reaction and require specialized medical care and support [7,8,9].

Moreover, people who manifest severe symptoms affecting the airways may need the support of mechanical ventilation, exposing them to infections other than COVID-19, such as pneumonia. People suffering from COVID-19 are also at higher risk of stroke or heart attack. In addition, some patients may show symptoms related to the nervous system, such as transient changes in personality or alertness levels [10,11,12].

In the general population, there are specific groups of people who are more prone to be infected and develop the disease. Some criteria of this discrimination are age (especially people over 60 years old), pregnancy and underlying diseases such as obesity, hypertension, diabetes, cardiovascular disease, long-term diseases affecting the lungs and airways, and diseases related to a burdened immune system. Symptoms in children tend to be milder than in adults. However, children remain carriers of the virus, and some of them become seriously ill [13,14].

Vaccination is the most effective way to prevent the severe complications of COVID-19 combined with measures such as wearing a mask, maintaining physical distance, good indoor ventilation and regular hand washing (soap or alcohol-based), which help to avoid the transfer of the virus from the hands to the body through the eyes, nose or mouth. Vaccinated people are less likely to manifest severe symptoms of the disease or to be hospitalized. That is why public health officials are urging all eligible people to get fully immunized against COVID-19 [15,16,17].

Drugs are now becoming available to treat COVID-19 that directly target the virus. They are mainly used to prevent severe manifestations of the disease in high-risk groups. The primary treatment for most patients with severe disease remains supportive care, such as the use of oxygen therapy and the management of fluid levels. In addition, the global research community has emphasized the development and mass production of effective drugs and vaccines [18,19,20].

Machine Learning is a field of Artificial Intelligence (AI) that deals with the study and construction of computational algorithms that can automatically be improved through experience. Machine Learning algorithms create one model based on sample data, also known as training data, in order to make predictions or make decisions without being explicitly programmed on how to do it. Such algorithms have a field of application in various sectors. Machine Learning now has a significant contribution in the field of medicine for the prediction of various diseases and the early diagnosis of several chronic conditions such as diabetes (as classification [21,22] or times-series task for continuous glucose values forecasting [23,24]), high blood pressure (hypertension) [25,26], cholesterol [27,28], chronic obstructive pulmonary disease (COPD) [29], stroke [30], cardiovascular diseases (CVDs) [31], acute liver failure (ALF) [32], acute lymphoblastic leukemia [33], sleep disorders [34,35], hepatitis C [36], lung cancer [37], chronic kidney disease (CKD) [38], etc.

In this scientific article, following a supervised learning procedure, an ML-based framework will be described and analyzed in order to identify early-stage symptoms of SARS-CoV-2 occurrence. The key aspects of the adopted methodology are the following:

- Firstly, a data balancing approach is applied to address the non-uniform distribution of the samples in two classes, and thus, design effective classifiers. For this purpose, we exploited the Synthetic Minority Oversampling Technique (SMOTE) [39]. This method randomly selects instances of the whole data and creates new samples based on the K-Nearest Neighbor.
- Secondly, a features analysis is made in order to rank their importance by selecting three different methods. In addition, we measure nominal features’ frequency of occurrence in order to identify their relatedness with the SARS-CoV-2 class.
- Thirdly, the performance of various ML models’ performance is evaluated and compared in terms of Accuracy, Precision, Recall, F-Measure, and AUC. All metrics show that the stacking ensemble method prevailed over the other models; thus it is the main proposition of this analysis.
- Finally, a comparison with a published work on the same dataset and features that we relied on is performed, showing the superiority of our ML models in terms of Accuracy and AUC.

The following sections of this research work are formulated as follows. Section 2 describes the dataset we relied on and analyzes the adopted process. Furthermore, in Section 3, we present and discuss the research outcomes. Section 4 presents some works that exploit the ML models and techniques in order to identify early-stage symptoms of SARS-CoV-2 and COVID-19 occurrence. Finally, Section 5 summarizes the submitted article and sets future directions.

## 2. Materials and Methods

### 2.1. Dataset Description

In order to evaluate the ML models, we relied on a publicly available dataset [40]. The specific dataset includes 6512 participants, of which the number of men is 3367 (51.7%) and women 3145 (48.3%). The target class is SARS-CoV-2, which indicates if the participant is positive to the SARS-CoV-2 virus or not. The number of participants who were diagnosed positive for SARS-CoV-2 virus is 1572 (24.1%). The description of the dataset’s characteristics is detailed in Table 1.

### 2.2. Data Preprocessing

On the dataset [40] we experimented with, we applied SMOTE, an oversampling technique for increasing the number of cases in the dataset in a balanced way, and the synthetic samples [52] are generated for the minority class. SMOTE is based on the K-Nearest Neighbors model with K equal to 5 [53]. The instances in the SARS-CoV-2 class are oversampled such that the subjects in the two classes are uniformly distributed. After the implementation of SMOTE (see Algorithm 1), the number of participants is 9880. Now, the dataset is balanced, and the target class SARS-CoV-2 includes 4940 SARS-CoV-2 Positive and 4940 Non-SARS-CoV-2 Positive instances.
**Algorithm 1:** SMOTE**Input**: *M* (number of samples in the minority class), *N* (% ratio of synthetic minority samples for class balancing), *K* (number of nearest neighbors), $s_{syn}$ synthetic instance;Choose randomly a subset S of the minority class data of size $S=\frac{N}{100}M$ (synthetic samples in the minority class) such that the class labels are uniformly distributed; **for all**$s_{i}\in S$**do** (1) Find the *K* nearest neighbors; (2) Randomly select one of KNNs, called ${\hat{s}}_{i}$; (3) Calculate the distance $d_{i,k}={\hat{s}}_{i}-s_{i}$ between the randomly selected NN ${\hat{s}}_{i}$ and the instance $s_{i}$; (4) The new synthetic instance is generated as $s_{syn}=s_{i}+\delta d_{i,k}$ (where δ=rand(0,1) is a random number between 0 and 1);**end for**Repeat steps number 2–4 until the desired proportion of minority class is met.

### 2.3. Features Analysis

The features analysis will move into two axes, including participants’ prevalence per feature in terms of the target class and importance evaluation.

**Figure 1:**
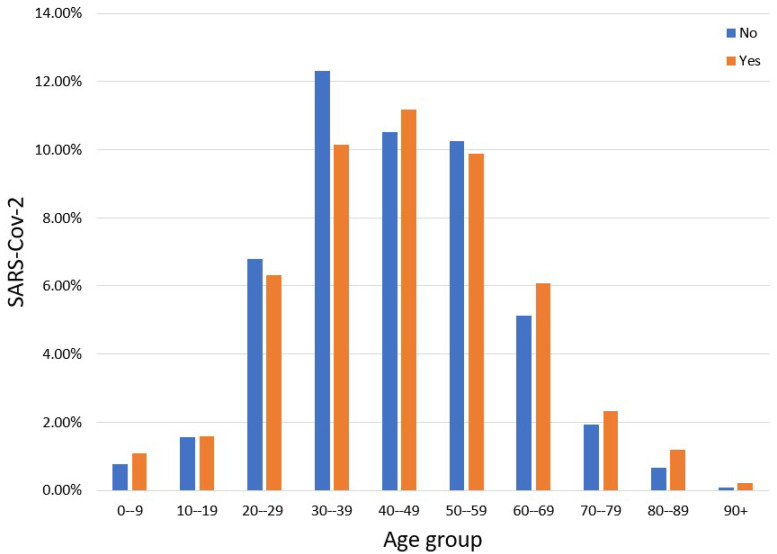
Percentage distribution of participants per age group.

Table 2 shows the percentage frequency of occurrence of participants’ in all features in terms of the two states of the class label. In addition, the mean age of participants in the balanced data is 44.39, and the standard deviation is 14.46 years old. Their prevalence, in each age group per class label (No, Yes), is shown in Figure 1. As for gender, men suffer from SARS-CoV-2 10% more than women. In addition, fever, cough and lung infection are the most frequently occurring symptoms in the class ‘Yes’, although these ones are not solely related to SARS-CoV-2.

For their importance ranking, we employed two methods, Information Gain and Random Forest. The respective outcomes are captured in Table 3.

The InfoGain [54] estimates the worth of an attribute *V* by measuring the information gain with respect to the class variable *C* as InfoGain(C,V) = H(C) − H(C|V). The first term defines the entropy of the class variable *C* which can be determined as $H\left(C\right)\;=\;-\sum_{c\in C}p\left(c\right)log_{2}\left(p\left(c\right)\right)$, where p(c) is the probability of c∈C = {0,1}, respectively. In addition, the second term $H\left(C|V\right)\;=\;-\sum_{v\in V}p\left(v\right)\sum_{c\in C}p\left(c|v\right)log2\left(p\left(c|v\right)\right)$ is the conditional entropy of the class variable *C* given an attribute *V*, where p(v) is the probability of value *v* and p(c|v) is the conditional probability of class value *c* given *v*.

In the Random Forest method, the purity of the leaves captures the feature importance. Feature importance is averaged among all the trees and normalized such that the sum of the importance scores is equal to 1 [55]. Their importance increases with the increase in leaf purity.

Observing the ranking scores in Table 3, we see that both methods identify cough as the feature with the highest importance, while fever and lung_infection follow reverse ranking order comparing the two methods. Additionally, pneumonia, runny_nose and travel_history are the next most important features whose order differs among the InfoGain and Random Forest. In addition, diarrhea and muscle_soreness have been listed last in order with a rank close to zero. For the models’ training and testing, all features were considered.

### 2.4. Machine Learning Models

In this submission, we experimented with various ML models to uncover which one outperforms the rest by evaluating their prediction performance. Specifically, we focused on Naive Bayes (NB) [56] and Logistic Regression (LR) [57] models, which are probabilistic classifiers. Furthermore, we used the well-known kernel-based (linear, non-linear) classifier Support Vector Machine (SVM) [58] and the Sequential Minimal Optimization (SMO) [59] that quickly solves the SVM Quadratic Programming problem. In addition, Stochastic Gradient Descent (SGD) [60] learning of a linear classifier under SVM convex loss function was applied.

Moreover, we used Decision-Tree-based models such as Hoeffding Tree (VFDT) [61], J48 [62], Random Tree (RT) [63], XGBoost [64], and Gradient Boosting Machine (GBM) [65]. From Ensemble ML algorithms [66], Bagging [67], Random Forest (RF) [68], Rotation Forest (RotF) [69], AdaBoostM1 [70], Voting [71], and Stacking [72] were exploited. Finally, a simple Artificial Neural Network (ANN) [73], the Multi-Layer Perceptron (MLP) [74] and K-Nearest Neighbors (kNN) [53], a distance-based classifier, were evaluated.

### 2.5. Evaluation Metrics

To evaluate the performance of the ML models, we utilized the most common metrics including Accuracy, Recall, Precision, F-Measure, and AUC [75]. The definition of these metrics is based on the Confusion Matrix, which consists of the elements True Positive (TP), True Negative (TN), False Positive (FP) and False Negative (FN). More specifically, they are determined as follows:

- Accuracy: It is the percentage of correct predictions for the test data.
  (1) $$\begin{array}{c} \mathrm{Accuracy}=\frac{\mathrm{TN}+\mathrm{TP}}{\mathrm{TN}+\mathrm{TP}+\mathrm{FN}+\mathrm{FP}} \end{array}$$
- Recall: It corresponds to the proportion of participants diagnosed with SARS-CoV-2 and correctly considered positive relative to all positive participants.
  (2) $$\begin{array}{c} \mathrm{Recall}=\frac{\mathrm{TP}}{\mathrm{TP}+\mathrm{FN}} \end{array}$$
- Precision: It indicates how many of those who were positive to SARS-CoV-2 belong to this class.
  (3) $$\begin{array}{c} \mathrm{Precision}=\frac{\mathrm{TP}}{\mathrm{TP}+\mathrm{FP}} \end{array}$$
- F-Measure: It is the harmonic mean of the Precision and Recall and sums up the predictive performance of a model.
  (4) $$\begin{array}{c} F-\mathrm{Measure}=2\frac{\mathrm{Precision}\cdot \mathrm{Recall}}{\mathrm{Precision}+\mathrm{Recall}} \end{array}$$
- The Area Under the Curve (AUC) is the measure of the ability of a classifier to distinguish between classes. It is a metric that varies in [0, 1].

## 3. Results

### 3.1. Experiments Setup

In order to evaluate the classification models, we worked on the Weka tool that offers an Environment for Knowledge Analysis [76]. Weka is an open-access software that comprises a set of ML algorithms for various data mining tasks. It contains tools for data preparation, classification, regression, clustering, association rules mining, and visualization.

The computer system we experimented with has the following specifications: 11th Gen Intel(R) Core(TM) i7-1165G7 @ 2.80 GHz, RAM 16 GB, Windows 11 Home, 64-bit OS and x64 processor. For the experiment results, 10-fold cross-validation was applied to measure the effectiveness of the models on the balanced dataset of 9880 cases after SMOTE. In Table 4, we depict the optimal parameters’ settings of the ML models acquired such that the Recall and AUC metrics are as high as possible. The outcomes that will be presented in the following were derived under such an assumption. In addition, the SMO, GBM, AdaBoostM1 and RotF models had as base classifiers the RF. Concerning the ensemble methods, the Stacking model had the RF and J48 models as base classifiers and as meta classifier the LR. The Bagging model had as base classifier the RF, and finally, the Voting model had as base classifiers the RF and J48 models.

### 3.2. Performance Evaluation

The performance assessment of the classifiers we experimented with was performed after SMOTE and 10-fold cross-validation. Plenty of well-known ML models including NB, SVM, LR, ANN, KNN, SGD, SMO, VFDT, J48, RF, RT, XGBoost, GBM, RotF, AdaBoostM1, Stacking, Bagging, and Voting were exploited and compared to identify early-stage symptoms of SARS-CoV-2 occurrence. The ML models’ assessment was accomplished with the aid of Accuracy, Precision, Recall, F-Measure and AUC metrics.

In Table 5, we provide a performance evaluation of ML models after SMOTE with 10-fold cross-validation. The lowest performance of the models we relied on is presented by the SVM and SMO with an Accuracy, Recall, F-Measure and AUC equal to 88.3%, and Precision of 88.5%. The KNN, RF, RT, XGBoost, GBM, RotF, AdaBoostM1, Stacking, Bagging, and Voting models show similar accuracy rates with rates greater than 90.4%. Finally, the Stacking model outperforms the others, achieving an Accuracy, Precision, Recall and F-Measure equal to 90.9% and an AUC of 96.4%, which constitutes our main proposition for this submission.

**Figure 2:**
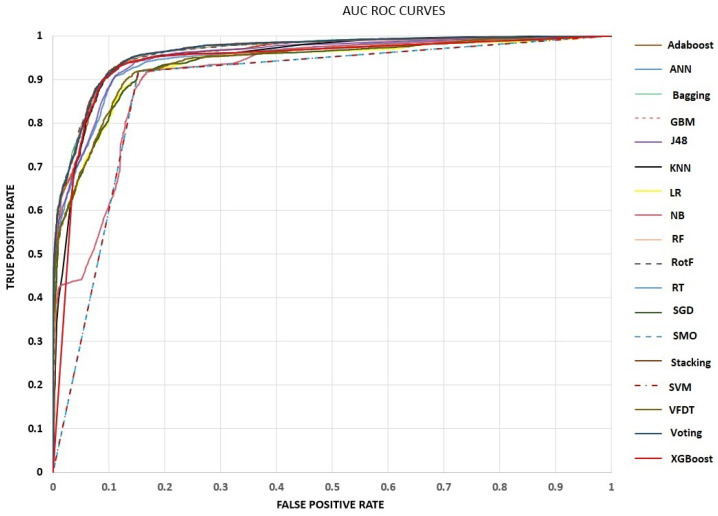
AUC ROC Curves.

In terms of the AUC metric, our proposed models achieved percentages greater than 91.1%, except for SVM and SMO, which obtained a percentage of 88.3%. As we can see from Figure 2, the performance of RotF, Stacking, Bagging, and Voting models is almost identical, which is also confirmed and numerically.

Next, we isolate study [77] where the authors experimented with the same dataset [40] and features we relied on. Based on this work, in Table 6, we present its ML models’ performance in terms of Accuracy and AUC from which it is shown that our proposed models outperformed (more or less) in both metrics. As a final note, we see that the proposed models are characterized by high separation ability (according to AUC) and high prediction accuracy of an unclassified instance.

## 4. Discussion

In this section, we will carry out a brief discussion of works that have fields of application AI/ML techniques and models to identify early-stage symptoms of SARS-CoV-2 infected patients and prediction of the disease occurrence. AI tools are a solution for patient screening where PCR-based diagnostic tools [78] are limited.

First, in [79], the authors used the blood profile of the potential patient to train Logistic Regression, Glint, Random Forest and Artificial Neural Network for patients in regular ward testing and not admitted to hospital (community) testing for SARS-CoV-2 positive. Sensitivity, Specificity, Accuracy and AUC were utilized to validate the models’ performance. Second, [80] demographic and several routine laboratory measurements were used to develop promising ML models (such as Logistic Regression, Decision Trees, Random Forest and Gradient Boosting Decision Tree) that can provide an accurate prediction of SARS-CoV-2 infection status.

In addition, in [81], the authors analyze from various perspectives (such as prevention of viral spread, care management, vaccines, etc.) the contribution of machine learning approaches to SARS-CoV-2. Diagnosis and patient screening are the most commonly occurred applications of AI/ML to SARS-CoV-2 based on medical images such as computed tomography, X-ray and other clinical measurements by applying Convolutional Neural Networks, Support Vector Machine, Random Forest, and MultiLayer Perceptron. In [82], the XGBoost model was applied with an accuracy higher than 90% in order to investigate the probability of mortality of an individual SARS-CoV-2 patient.

The researchers in [83] suggested the combination of antibody responses to multiple antigens to identify individuals with previous SARS-CoV-2 infection using ML classifiers trained with multiplex data. The Random Forests algorithm was selected due to its superiority over other classifiers such as Logistic Regression.

Furthermore, the research study in [84] employed ML for a different purpose against COVID-19. In particular, they combined in silico methods such as virtual drug screening, molecular docking and supervised machine learning algorithms to identify novel drug candidates.

Moreover, the authors in [85] developed a web platform to estimate anti-SARS-CoV-2 activities and identify active molecules for COVID-19 treatment with the support of machine learning. Several prediction models were developed for eleven models of viral entry, replication, live and in vitro infectivity, and human cell toxicity, employing three categories of features (chemical fingerprints, physicochemical and topological pharmacophore descriptors) and 22 distinct ML classifiers.

In [86], ML models were trained and validated using basic blood test measurements which were compared to reference RT-PCR testing to predict COVID-19 infection status. In addition, the authors explored the improvement provided in the basic clinical model by the use of chest radiographs. The evaluation was made via the employment of AUC, Sensitivity, and Specificity. The categorical gradient boosting model was trained to classify whether the patient has COVID-19 and other viral pneumonia, bacterial pneumonia or non-pneumonia.

Contrary to the abovementioned studies, in this work, we considered non-biochemical data acquired by a non-invasive process. Actually, it is a dataset that captures the most relevant symptoms of SARS-CoV-2. From this point of view, clinical features are considered to train and test the ML models. The authors in [77], exploiting the same dataset [40] that this study considered, applied XGBoost, Gradient Boosting Machine, Support Vector Machine, Random Forest and Decision Tree in order to identify early-stage symptoms of SARS-CoV-2 infected patients. Their experimental results showed that the XGBoost algorithm outperforms the other ones in terms of Accuracy and AUC. However, in our study, more efficient classifiers were selected with an emphasis on ensemble ones. We assumed 10-fold cross-validation on a balanced dataset and not percentage data splitting (as the comparing study considered) and emphasized ensemble models which the previous work did not apply along with a graphical illustration of AUC curves. Finally, comparing the performance of these models with the ones applied here, our trained and tested classifiers prevailed in both metrics.

## 5. Conclusions

The coronavirus disease (COVID-19) pandemic was caused by the SARS-CoV-2 virus. The virus was first reported in the Wuhan region of China at the end of December 2019. In severe cases, pneumonia, acute respiratory distress syndrome, multiple organ failure or even death may occur from the virus. Machine Learning now has a significant contribution in the field of medicine for the prediction of various diseases and the early diagnosis of several chronic conditions.

In the present article, a supervised ML-based framework is presented and analyzed in order to identify early-stage symptoms of SARS-CoV-2 occurrence. For this purpose, a variety of well-known ML models and techniques were exploited to confirm the most effective model in terms of Accuracy, Precision, Recall, F-Measure and AUC. The experimental results showed that the Stacking model outperformed the others, achieving an Accuracy, Precision, Recall and F-Measure equal to 90.9% and an AUC of 96.4% after SMOTE with 10-fold cross-validation. Our proposed models prevailed in relation to the compared models of research work [77].

At this point, we have to mention some limitations of this submission. Firstly, we relied on a publicly available dataset from Kaggle [40] to evaluate the performance of the models from an ML technical point of view. In a different dataset from Electronic Health Records (EHR), richer features could help to draw more useful medical conclusions. However, it should be noted that medical data is sensitive, and access is restricted or hardly gained. In addition, since the size of the dataset was not extensive enough, in future studies, using much larger datasets could further improve the predictive accuracy.

In a future direction of the current study, we aim to design and evaluate each model by dividing the initial data into subsets based on the age groups or the gender type of the subjects. Such an approach will help to analyze the relationship between COVID-19 and patient age and gender. In addition, since the size of the dataset was not extensive enough, in future study, our purpose is to experiment with larger datasets that could further improve the predictive accuracy. Finally, we plan to expand the ML framework by applying Deep Learning models such as Convolution Neural Networks and Long Short-Term Memory Networks and comparing their predictive ability in terms of the aforementioned metrics.

## Figures and Tables

**Table 1:** Dataset Description.

Feature	Type	Description
gender [41]	nominal	This feature illustrates the participants’ gender.
age (years) [42]	numeric	The age range of the participants is 0–96 years.
fever [43]	nominal	If the participant has body temperature greater than 38 °C.
cough [44]	nominal	If the participant has a severe cough.
runny_nose [45]	nominal	If the participant has a runny nose.
muscle_soreness [46]	nominal	If the participant has muscle soreness.
pneumonia [47]	nominal	If the participant has pneumonia.
diarrhea [48]	nominal	If the participant has diarrhea.
lung_infection [49]	nominal	If the participant has lung infection.
travel_history [50]	nominal	If the participant has travel history.
isolation_history [51]	nominal	If the participant received isolation treatment in designated hospitals.
SARS-CoV-2	nominal	This feature illustrates if the participant is positive to SARS-CoV-2.

**Table 2:** Participants distribution per feature value and class label in the balanced dataset after SMOTE.

		SARS-CoV-2 Class Label	
Feature	Value	No	Yes
gender	female	24.05%	20.09%
	male	25.95%	29.91%
fever	No	35.50%	9.93%
	Yes	14.50%	40.07%
cough	No	42.03%	11.12%
	Yes	7.97%	38.88%
runny_nose	No	49.52%	34.30%
	Yes	0.48%	15.70%
muscle_soreness	No	49.81%	49.93%
	Yes	0.19%	0.07%
pneumonia	No	50.00%	34.53%
	Yes	0.00%	15.47%
diarrhea	No	49.69%	49.94%
	Yes	0.31%	0.06%
lung_infection	No	49.30%	25.47%
	Yes	0.70%	24.53%
travel_history	No	12.98%	31.81%
	Yes	37.02%	18.19%
isolation_treatment	No	41.88%	34.63%
	Yes	8.12%	15.37%

**Table 3:** Features importance evaluation using Information Gain and Random Forest.

InfoGain		Random Forest	
Feature	Rank	Feature	Rank
cough	0.298465	cough	0.30911
lung_infection	0.262035	fever	0.25567
fever	0.199997	lung_infection	0.23836
pneumonia	0.175181	travel_history	0.18836
runny_nose	0.15083	pneumonia	0.15466
travel_history	0.106258	runny_nose	0.15223
isolation_treatment	0.021412	age_year	0.0874
gender	0.004587	isolation_treatment	0.07257
age_year	0.004074	gender	0.03957
diarrhea	0.001355	diarrhea	0.00253
muscle_soreness	0.000421	muscle_soreness	0.00121

**Table 4:** Machine Learning Models’ Parameters Settings.

Model	Parameters
**NB**	useKernelEstimator: False useSupervisedDiscretization: True
**SVM**	eps = 0.001, gamma = 0.0 kernel type: linear, loss = 0.1
**LR**	ridge = $10^{-8}$ useConjugateGradientDescent: True
**ANN**	hidden layers: ‘a’ learning rate = 0.1 momentum = 0.2 training time = 200
**SGD**	epochs: 500 loss function: Log loss (logistic regression)
**SMO**	calibrator: Random Forest kernel: PolyKernel
**KNN**	K=1 Search Algorithm: LinearNNSearch Euclidean cross-validate = True
**VFDT**	leaf Prediction Strategy: Naive Bayes split criterion: Gini split
**J48**	reducedErrorPruning: False savelnstanceData: True useMDLCorrection: True, subtreeRaising: True binarySplits = True, collapseTree = True
**RF**	breakTiesRadomly: True numIterations = 100, numFeatures = 0 StoreOutOfBagPredictions: True
**RT**	maxDepth = 0, minNum = 1.0 minVarianceProp = 0.001
**XGBoost**	batchSize: 100 numDecimalPlaces: 2
**GBM**	classifier: Random Forest useEstimatePrior: True useResampling: True
**AdaBoostM1**	classifier: Random Forest, resume: True useResampling: True
**RotF**	classifier: Random Forest NumberOfGroups: True ProjectionFilter: PrincipalComponents
**Stacking**	classifiers: Random Forest and J48 metaClassifier: Logistic Regression numFolds = 10
**Bagging**	classifiers: Random Forest PrintClassifiers: True StoreOutOfBagPredictions: True
**Voting**	classifiers: Random Forest and J48 CombinationRule: AverageOfProbabilities

**Table 5:** Performance evaluation of ML models after SMOTE with 10-fold cross-validation.

	Accuracy	Precision	Recall	F-Measure	AUC
**NB**	0.871	0.872	0.871	0.871	0.911
**SVM**	0.883	0.885	0.883	0.883	0.883
**LR**	0.885	0.885	0.885	0.885	0.933
**ANN**	0.898	0.898	0.898	0.898	0.947
**SGD**	0.886	0.887	0.886	0.886	0.934
**SMO**	0.883	0.885	0.883	0.883	0.883
**KNN**	0.904	0.904	0.904	0.904	0.951
**VFDT**	0.885	0.886	0.885	0.885	0.939
**J48**	0.898	0.898	0.898	0.898	0.953
**RF**	0.906	0.907	0.906	0.906	0.960
**RT**	0.904	0.904	0.904	0.904	0.941
**XGBoost**	0.905	0.905	0.905	0.905	0.941
**GBM**	0.904	0.904	0.904	0.904	0.958
**AdaBoostM1**	0.905	0.905	0.905	0.905	0.956
**RotF**	0.907	0.907	0.907	0.907	0.962
**Stacking**	0.909	0.909	0.909	0.909	0.964
**Bagging**	0.905	0.906	0.905	0.905	0.963
**Voting**	0.907	0.908	0.907	0.907	0.963

**Table 6:** Comparison of our ML models with [77] in terms of Accuracy and AUC.

	Accuracy		AUC	
	Our Models	[77]	Our Models	[77]
**XGBoost**	0.905	0.880	0.941	0.850
**GBM**	0.904	0.860	0.958	0.880
**SVM**	0.883	0.860	0.883	0.880
**RF**	0.906	0.860	0.960	0.830
**DT**	0.885	0.860	0.939	0.820
